# Opportunities to Address Health Disparities in Suicidality for Sexual and Gender Minority Youth in Public Systems

**DOI:** 10.1002/mhs2.100

**Published:** 2024-12-19

**Authors:** Dana M. Prince, Megan S. Schuler, Katherine Lewis, Michelle R. Munson, Aaron J. Blashill, Peter S. Hovmand

**Affiliations:** 1 Mandel School of Applied Social Sciences, Case Western Reserve University, Cleveland, Ohio, USA; 2 RAND, Santa Monica, California, USA; 3 Begun Center for Violence Prevention and Education, Mandel School of Applied Social Sciences, Case Western Reserve University, Cleveland, Ohio, USA; 4 Silver School of Social Work, New York University, New York, New York, USA; 5 Department of Psychology, San Diego State University, San Diego, California, USA; 6 Center for Community Health Integration, Case Western Reserve University School of Medicine, Cleveland, Ohio, USA; 7 Biomedical Engineering, Case School of Engineering, Case Western Reserve University, Cleveland, Ohio, USA

**Keywords:** child welfare, implementation science, juvenile justice, sexual and gender minority, suicide

## 1 | Introduction

The prevention of self-injurious thoughts and behaviors (SITB) among youth continues to be a public health imperative. In the general population, suicide is the second-leading cause of death for ages 10–24 (Ruch et al. 2019). However, specific subgroups of youth are at significantly greater risk of SITB. Public systems involvement, LGBTQ+ status, and Black and/or Latinx youth are at elevated risk. Moreover, LGBTQ+ and Black/Latinx youth—and those who are both—are disproportionately overrepresented in the child welfare and juvenile justice systems. Child welfare and juvenile justice involved youth have approximately three times greater risk for suicide ideation, attempts, and completions (i.e., self-injurious thoughts and behaviors) than non-systems-involved youth (Agencies 2013; Casiano et al. 2013; Evans et al. 2017; Gallagher and Dobrin 2005; Gray et al. 2002; Hayes 2009; Katz et al. 2011; Scott, Underwood, and Lamis 2015; Vinnerljung, Hjern, and Lindblad 2006). Sexual and Gender Minority (SGM) youth in the general population have two to four times the risk of SITB compared to their heterosexual, cisgender peers (Luk et al.2021; Nock et al. 2013; Perez-Brumer et al. 2017). Notably, SGM youth are disproportionately overrepresented in child welfare and juvenile justice, with estimates ranging from 16% to 32% (Grant et al. 2011; Majd, Marksamer, and Reyes 2009; Matarese et al. 2021; Wilson and Bouton 2022; Wilson and Kastanis 2018; Wilson et al. 2017) compared to 2%–8% in the general population (Conron et al. 2014). In sum, the risk of SITB for SGM youth who are involved with public systems is compounded (Dettlaff et al. 2018; Johns et al. 2020; Scannapieco, Painter, and Blau 2018).

Child welfare and juvenile justice systems can screen, assess, and refer to treatment youth who may not otherwise access services (Casiano et al. 2013; Gallagher and Dobrin 2005; Gray et al. 2002). The unique needs of system-involved SGM youth have been largely ignored, with few child welfare and juvenile justice jurisdictions systematically identifying SGM youth or providing SGM-affirming care (Busby et al. 2020; Call, Challa, and Telingator 2021; Evans et al. 2017; Rider et al. 2019). There is a clear and urgent need for system-level interventions to provide SGM youth with equitable care to improve SITB and other behavioral health outcomes. In this paper, we provide a conceptual framework that can guide system-level research in this area, as well as highlighting several key knowledge gaps and research opportunities.

### 1.1 | Conceptual Model

**FIGURE 1 |:**
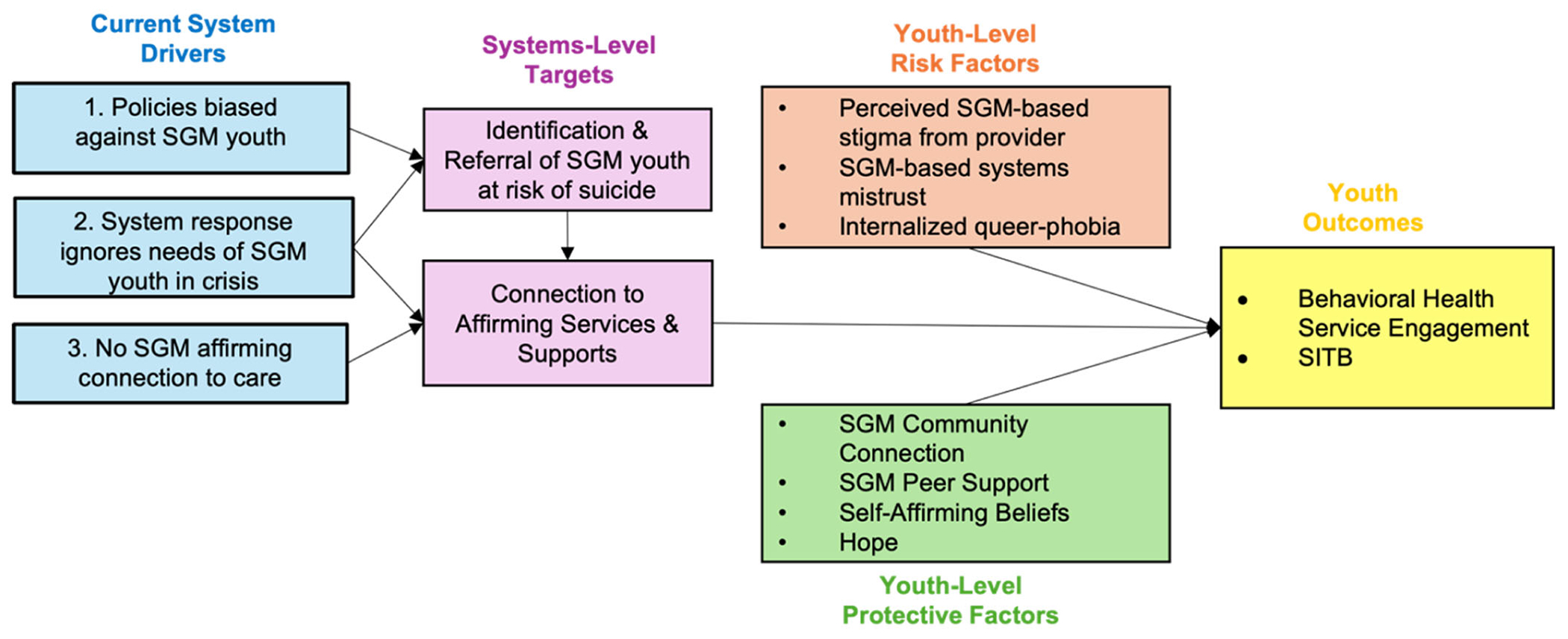
Conceptual model.

The Minority Stress Framework is a key theoretical framework that can inform research and intervention work regarding SGM youth (Lick, Durso, and Johnson 2013; Meyer 2003; Meyer and Frost 2013; Testa et al. 2015). Minority stress theory identifies SGM-specific stressors across levels (i.e., institutional, interpersonal, intrapersonal) as drivers of mental health disparities for SGM groups (Matsuno 2019; Mitchell et al. 2015; Price-Feeney, Green, and Dorison 2020). Despite the broad recognition that structural and systems-level factors drive health disparities (see e.g., Hatzenbuehler, 2016 [Hatzenbuehler 2016]), the application of minority stress theory has primarily focused on individual-level stressors and outcomes. However, our previous conceptual work (Prince et al. 2022) shows how unique stressors within the child welfare system (e.g., biased policy, [Bellamy, Schmutte, and Davidson 2017; Chinman et al. 2018; Gopalan et al. 2017; Lick, Durso, and Johnson 2013] SGM-based abuse, [McGeough and Sterzing 2018; Sterzing et al. 2017] lack of affirmative mental health care [de Lange et al. 2022; Taliaferro et al. 2019; Taliaferro and Muehlenkamp 2017; Worrell et al. 2022]) contribute to the disproportionate burden of behavioral health disorders among SGM youth (Tebbe and Moradi 2016; Testa et al. 2017; Strauss et al. 2020; Worrell et al. 2022). Additionally, our previous research demonstrates how factors from the Minority Stress Framework related to disconnection, avoidance or delay of behavioral health treatment (Gillani et al. 2024). The conceptual framework shown in Figure 1 identifies both system-level and individual-level drivers and that could serve more broadly for multi-level intervention research with this population.

### 1.2 | System Driver 1

Currently, biased policies within some child welfare and juvenile justice systems impedes the collection of sexual and gender minority youth data (Bellamy, Schmutte, and Davidson 2017; Chinman et al. 2017, 2018; Gopalan et al. 2017). SGM youth may be uncomfortable disclosing their identities due to previous system interactions (e.g., being “outed” or having personal information shared with unknown parties) (de Lange et al. 2022). When screening youth for SGM-status, an affirming/normalizing approach is needed, yet this is not current practice in most systems (Worrell et al. 2022). Clear guidelines and training for system workers about where, when, and how to ask youth these questions are needed.

### 1.3 | System Driver 2

Without safe identification procedures, systems fail to identify a key factor—SGM status—related to youth SITB risk, and thus are unable to provide targeted and tailored interventions, such as SGM-affirming care, to SGM youth at risk for SITB.

### 1.4 | System Driver 3

Although child welfare and juvenile justice systems do systematically screen youth for SITB and refer youth to behavioral treatment, the treatment opportunities provided are not necessarily SGM-affirming. Again, this is a critically missed opportunity, as prior research has shown that lack of access to SGM-affirming mental health providers and client-perceived SGM-based bias within care settings leads to worse engagement and avoidance or delay of services (Powell and Cochran 2021).

## 2 | Gaps and Opportunities

### 2.1 | System-Level Identification of SGM Youth

Currently, child welfare agencies are not required to collect sexual identity or gender identity information on youth in their care (Wilber 2013). Yet, rates of depression, suicide ideation, suicide attempts, and hospitalizations among SGM youth in foster care far exceed those of their heterosexual and cisgender peers (Dettlaff et al. 2018; Prince et al. 2024; Wilson and Kastanis 2015). Unsupportive and even hostile cultures towards SGM youth within the child welfare system may contribute to their poor health outcomes (Fish et al. 2019). No studies to date of juvenile justice-involved youth have examined SITB prevalence among SGM youth due to the lack of routinized collection of SGM data in most juvenile justice systems.

### 2.2 | SGM Affirming Services and Supports

In many child welfare and juvenile justice systems across the U.S., the current suicide risk safety net fails to adequately identify and connect SGM youth to appropriate care. Sexual and gender minority youth, particularly those with public system-involvement, experience barriers to engagement with treatment that supports their health and well-being (Hong, Espelage, and Kral 2011; Opara et al. 2020; Scannapieco, Painter, and Blau 2018). These youth may experience discriminatory and traumatizing interactions with healthcare and social service providers, leading to delays in accessing mental health care, disengagement from services and exacerbation of negative health outcomes (Kodish et al. 2020; Oliffe et al. 2019). Improving outcomes for SGM youth requires tailored services, such as SGM-affirming care (Ehrensaft et al. 2018; Ellis 2020). SGM-affirming care has been shown to improve outcomes for SGM youth compared to traditional services.21, (Call, Challa, and Telingator 2021; Sapiro and Ward 2020) However, availability of SGM-affirming services and supports is uneven in public systems serving youth. We highlight formal and informal peer support (e.g., individuals with shared lived experience) as one high promise and low-resource avenue to improve outcomes for SGM youth in systems.

### 2.3 | Peer Support

A sharp increase in U.S. youth mental health problems (National Academies of Sciences, Engineering, and Medicine 2022) coupled with the national shortage in child behavioral health clinicians (National Academies of Sciences, Engineering, and Medicine 2017) has identified peer providers as a cost- and impact-effective solution (National Academies of Sciences, Engineering, and Medicine 2017). Among LGBTQ+ populations, greater social support (e.g., friends, peers, fictive kin, and chosen family) is associated with decreased suicide risk (de Lange et al. 2022; Hatchel, Polanin, and Espelage 2021; Wilson and Cariola 2020). Research also indicates that members of the LGBTQ+ community express high interest in serving as peer supporters for others in the community, particularly concerning suicide risk (Worrell et al. 2022). Utilizing peers as part of suicide prevention interventions with LGBTQ+ populations is growing; however, research in this area is still nascent. For example, peer support is beginning to be integrated into evidence-based treatments to enhance client engagement and improve outcomes (Giovanelli et al. 2023). Peer Support Specialists (PSS), a paraprofessional with lived experience who provide mutual and structured support, have been found to be effective in improving client engagement in mental health services (Bellamy, Schmutte, and Davidson 2017; Chinman et al. 2018; Gopalan et al. 2017; Ojeda et al. 2021). Youth peer support services are already widely used across public systems for youth with mental and behavioral health needs including community-based mental health agencies, child welfare, and juvenile court (Gopalan et al. 2017). Despite the widespread usage of peers in behavioral health, research on effectiveness is lacking (Bellamy, Schmutte, and Davidson 2017). Future research should attend to the use of peer providers in behavioral health interventions, including for SGM youth and in public system environments.

### 2.4 | Recommended Research Strategies and Solutions

1. Historically, minority stress theory is limited by its focus on individual-level outcomes, and weaker conceptualization of structural and systems-level drivers of health disparities. Our application of minority stress is unique and well suited to address theorized and empirically based system-level and interpersonal-level drivers of minority stress.
2. System Science Methodology. Systematic review of the PSS literature show that implementation barriers are common; yet studies that use system dynamics and implementation science are sorely lacking (Chinman et al. 2017). Research in this area calls for attention to specific implementation strategies and the inclusion of persons with lived experience as part of the research team (Chinman et al. 2017). Hallmarks of a system dynamics approach include modeling of dynamic changes in system behavior over time, and conceptualization of nonlinear relationships, and dynamic feedback systems (e.g., reinforcing and balancing loops).
3. Persons with lived experience play a unique and impactful role in mental and behavioral health service delivery (Bochicchio et al. 2021). Especially among multiply marginalized and minoritized groups, like systems-involved sexual and gender minority youth of color, peer providers can effectively meet clients “where they are” and provide instrumental, emotional, and relational supports unique to their shared group memberships and experiences. More research is needed to investigate the processes and outcomes of peer support that are most effective. Principles of community-engaged research are one useful strategy to guide this work.

## 3 | Conclusion

Sexual and gender minority youth involved in public systems experience very pronounced disparities in terms of mental and behavioral health, yet remain underserved and understudied. Disparities are likely to become even more pronounced in the current sociopolitical reality. A surge of anti-LGBTQ bills have been introduced in the past 3 years, many targeting youth and transgender/gender diverse youth in particular, severely limiting access to gender affirming care (Gzesh et al. 2024). Structural- and systems-level drivers of health disparities must be addressed to influence long-term social change and advance health equity for marginalized SGMY.

## Data Availability

The authors have nothing to report.
